# Phytochemistry and Applications of *Cinnamomum camphora* Essential Oils

**DOI:** 10.3390/molecules27092695

**Published:** 2022-04-22

**Authors:** Sang-Hwan Lee, Dae-Shin Kim, Seong-Hee Park, Hyun Park

**Affiliations:** 1 Technical Research Institute, Mine Reclamation Corporation, Wonju 26464, Korea; 2 Hallasan Research Department, World Heritage Office, Jeju City 63143, Korea; kds3374@korea.kr; 3 Fine Korea Co., 70 Seonyu-ro, Yeongdeungpo-gu, Seoul 07294, Korea; indipark62@hanmail.net; 4 Department of Biotechnology, College of Life Sciences and Biotechnology, Korea University, Seoul 02841, Korea

**Keywords:** biological activity, camphor oil, *Cinnamomum camphora*, essential oil, phytochemistry

## Abstract

Camphor tree (*Cinnamomum camphora*) is an ornamental plant that has been cultivated for a long time to obtain wood or camphor. Furthermore, its essential oil can be used as an alternative medicine and is an important source of perfume. Camphor obtained from camphor trees has long been used as a treatment for various symptoms such as inflammation, infection, congestion, muscle pain, and irritation in various regions. The purpose of this literature review is to provide knowledge of the well-established, wide, and extensive applications of camphor both in traditional and modern applications. Despite many studies focused on the essential oil of the camphor tree, there is a lack of systematic studies of its extraction or separation. Besides, various components of camphor are not fully understood, and further research is needed on the medicinal effects of individual components of *C. camphor*. The genus *Cinnamomum* has crucial economic value and theoretical significance. However, further systematic reviews and investigative studies based on existing research are needed to promote the modernization process of traditional applications of camphor. For proper use of the essential oil of *C. camphora*, it is imperative to consider its possible effects on humans and the environment.

## 1. Introduction

Camphor tree (*Cinnamomum camphora*) is a member of the Lauraceae family, and is known to be native to India, China, and South Korea, and is now distributed in many other regions such as Australia and the Himalayas [1,2,3,4]. Camphor trees can reach up to several tens of meters (30–40 m) in height and 3 m in diameter and typically grow at 900–2500 m above sea level. The bark is yellow or brown and is vertically split. The leaves alternately have three to several distinct veins, and strong dormant buds surrounded by large, silky-like recesses [5].

This tree is one of the plant species that is highly valued in Asia in terms of its economy and culture, and large-scale cultivation has been established in China and Korea and has been designated and protected as a cultural property [6]. *C. camphora* is designated and protected as a natural monument in Jeju, Korea. KMoCST (Korea Ministry of Cultural Sports and Tourism) announced that it has traditionally been used as a medicinal plant and, in addition to its high biological value, it has high cultural value, providing a glimpse into traditional Asian life [7]. *C. camphora* is facing great pressure and threat, with an increased interest in its value leading to indiscriminate use and a sharp drop in numbers, which could lead to extinction if appropriate protective measures are not taken. To preserve and protect this endangered species, it is crucial to secure genetic diversity data. Maintenance of genetic diversity is essential to the long-term survival of the tree species, without which there may be a risk of its extinction because of a lack of adaptive ability [8]. Camphor trees have many applications in various fields, such as industry, cosmetics, pesticides, pharmaceuticals, timber, ornamental, and many cultural purposes stretching back thousands of years [6]. In the past, various parts of the camphor tree (leaves, stems and fruits) have been used to prepare essential oils and extracts with anti-inflammatory [9], or antifungal [10,11,12] properties, or to treat toothache [13], and spasmodic effects of circulatory and respiratory diseases. Camphor tree is used in the production of perfumes, creams, and balsamic ointments in the cosmetics industry [6,9] and as a treatment for muscle pain, inflammation, and rheumatism in the pharmaceutical sector [14,15,16,17]. The development of modern pharmaceutical technologies provides a theoretical background for the performance of *C. camphora*, which has been used in traditional medicine for a long time. The main research results so far are that the pharmacological effects are mainly derived from essential oils of *C. camphora*. Due to the development of technologies, essential oil can be extracted from various parts of *C. camphora* and components of essential oils exhibiting pharmacological effects can be separated and purified. Essential oils are mainly extracted by distillation.

Many different synthetic chemicals are produced and used in a wide range of applications; however, some synthetic agents may threaten our health if used at more than the recommended dosage [18]. Therefore, the use of essential oils obtained from plants is an attractive alternative to synthetic chemicals and additives for ensuring food safety, retention of their nutritional value and quality, and human health care. Some essential oil components extracted from various parts of camphor tree are found to have high antimicrobial activity and are certified as foodstuffs and utilized [19,20,21,22]. Moreover, the trend over recent decades toward the use of natural plant products in human medicine has resurrected interest in the use of essential oils as alternative medicines [23].

A bibliographic review of documents published between 2000 and 2021 on the subject was conducted. Literature searches with the following terms in English and Korean were carried out: “volatile compounds”; “functional uses” and “essential oil” associated with “*Cinnamomum camphora*”. The data search tools were used Google Scholar (https://scholar.google.com.br, accessed on 1 December 2021), Web of Science (https://www.web.of.sciencegov.br/, accessed on 1 December 2021), and PubMed (https://pubmed.ncbi.nlm.nih.gov/, accessed on 1 December 2021). This review was conducted to collect information related to the phytochemistry of the genus *Cinnamomum* and ultimately lay the foundation for future research and development. To this end, phytochemical studies of the essential oil content (EOC) are reviewed, followed by a brief overview of the traditional uses of *C. camphora*. Finally, the biological activity of *C. camphora* extracts and essential oils and their constituents is examined, focusing on the antimicrobial and antioxidant actions and application examples. In addition, the current existing limitations, and the potential role of recent advancements in science and technology to overcome these limitations with improved efficacy and worldwide applicability are discussed.

## 2. Phytochemistry of EOC

Advances in analysis and separation techniques have led to extensive research on the chemical composition and properties of essential oils extracted from fruits, stems and leaves, to identify chemical components and their biologically active properties [9,11,13,24]. This review focuses on extracting essential oil from the leaves of *C. camphora*, which are easy to secure. Using roots, stems, and barks may damage *C. camphora*, but this is not the case with leaves.

Camphor (*C. camphora*) has several chemical varieties, each with different essential oil compositions [11,13]. Depending on the main components of its leaf oil, *C. camphora* can be categorized into five chemotypes: isoborneol, camphor, 1,8-cineole, linalool, and borneol types [9,11,13,24,25,26] (Table 1).

Pragadheesh et al. [11] reported on the camphor type in which the distillates of *C. camphora* leaves contained approximately 74% camphor, and less than 5% overall was contributed by the remaining major components. In the borneol-type, Shi et al. [13] identified 11 kinds of monoterpenes, 5 kinds of sesquiterpenes, and 4 kinds of oxyterpenes, such as (+)-borneol (66.8%), 1,8-cineole (4.1%), camphor (0.8%), and α-terpineol (0.4%). The oxyterpenes constituted the dominant group, occupying 72.2% of the volatile oil from young leaves. The other compounds included monoterpenes (24.4%), mainly α-camphene, β-pinene, and β-myrcene, and sesquiterpenes (2.8%), such as *trans*-caryophyllene, α-humulene, and γ-elemene.

Stubbs et al. [26] detected an average of 49.8% 1,8-cineole in the leaf EOC of the cineole-type. Other major constituents were sabinene (average 16.6%) and citronellol (average 9.3%), and in both types, α-pinene was present at an average concentration of 4.5%. Xu et al. [24] reported that the main component of EOC was 1,8-cineole (53.5%), followed by β-terpinene (17.4%), α-terpineol (9.5%), and (1*R*)-α-pinene (4.7%), in addition to minor constituents.

Chen et al. [27] identified linalool (26.6%), 1,8-cineole (16.8%), α-terpineol (8.7%), isoborneol (8.1%), β-phellandrene (5.1%), and camphor (5.0%) as the main constituents in the leaf EOC. Satyal et al. [10] found that the leaf oil of *C. camphora* from Makwanpur (Nepal) was dominated by camphor (36.5%), camphene (11.7%), and limonene (9.0%), with lesser amounts of sabinene (6.3%) and β-pinene (6.3%), whereas the leaf oil of *C. camphora* from Kavre (Nepal) contained almost exclusively camphor (98.0%).

It should be noted that the composition and content ratio of EOC differ depending on the plant parts. Guo et al. [9] confirmed that 27 constituent were contained in the essential oil extracted from the bark compared to only 17 constituents in the essential oil extracted from the fruit. The main components of the bark essential oil were D-camphor (51.3%), 1,8-cineole (4.3%), α-terpineol (3.8%), and 3-methyl-2-butenoic acid, oct-3-en-2-yl ester (3.1%). Conversely, safrole (29.0%), D-camphor (28.1%), linalool (12.8%), and 1,8-cineole (5.3%) were the main constituents of the fruit essential oil. The components identified exclusively in the essential oil extracted from the bark were γ-terpinene, isoterpinolene, 1,3,8-*p*-menthatriene, terpinen-4-ol, α-terpineol, eugenol, β-cadinene, and α-cubebene.

The biosynthesis of secondary metabolites and the proportion of individual substances in essential oils vary due to environmental factors, such as seasonal variation, geographic changes, light availability, vegetation and microorganisms, and soil pH [28]. Therefore, an accurate understanding of the phytochemicals is essential for the use of the essential oil.

## 3. Traditional Uses of *Cinnamomum* *camphora*

Camphor is a natural product extracted from *C. camphora* and widely used in pharmaceuticals, industry, and environmental fields. Since long ago, camphor has been widely used in the East for various purposes. Traditional medicine utilizes camphor’s ability for resuscitation, heat clearance, and pain relief, and it is often used to recover from fever, convulsions, stroke, sputum fainting, sputum coma, laryngeal pain, mouth pain, anthrax, and bloodshot eyes [29]. *C. camphora* has long been prescribed in traditional medicines for the treatment of inflammation-related diseases such as rheumatism, and for sprains in Korea [30]. To briefly explain how to use it, approximately 30 to 50 g of the plant is consumed up to three times a day in the form of an extract prepared with hot water and lipidic food.

In Japan, camphor was used to make torch flames brighter by adding a small amount, and in India, camphor was used to burn incense in temples during religious ceremonies because it was not irritating to the eyes [13,31].

In Ayurveda, *C. camphora* was used to treat bronchitis, colds, diarrhea, dysentery, edema, flu, metabolism and heart disease, and in Greece, it was used as a head tonic and heart treatment [15].

Camphor has been widely used as a fragrance in cosmetics, a flavoring food additive, a preservative in confectionery goods, an insect repellent, a plasticizer, and an intermediate in the synthesis of aroma chemicals [31,32]. Furthermore, camphor was used as a fumigation agent during the outbreak of smallpox and the Black Death; the body was covered with rose water and camphor perfume when the body was buried [33].

## 4. Biological Activity

This section discusses some of the numerous biological activities of *C. camphora*, particularly its essential oil. Some of the key results and their implications are summarized in Table 2 and discussed in further detail in this section.

### 4.1. Antimicrobial Activities

The traditional use of *C. camphora* for antiseptic purposes can be attributed to the antimicrobial activity of its essential oil, which has demonstrated a broad range of antimicrobial activities against different pathogens [10,23,27,34]. Poudel et al. [23] reported that the wood EOC showed effective antibacterial activity against *Serratia marcescens*. Likewise, the combined leaf/branch/wood EOC exhibited effective antifungal activity against *Aspergillus niger* and *Aspergillus fumigatus*, and the leaf EOC showed good antifungal activity against *Trichophyton rubrum*, with a minimum inhibitory concentration (MIC) of 78.1 µg/mL (Table 2).

**Table 2:** Examples of applications of essential oil of *Cinnamomum* *camphora*.

Activity	Effects/Applications	Reference
Antimicrobial	Inhibits *Choanephora cucurbitarum*	[11]
	Inhibits *Serratia marcescens*, *Aspergillus niger*, *Aspergillus fumigatus* and *Trichophyton rubrum*	[23]
	Inhibits *Staphylococcus aureus*, *Enterococcus faecalis*, *Bacillus subtilis*, *Salmonella enterica* subsp. *enterica* serovar Gallinarum, and *Escherichia coli*	[27]
	Inhibits *Listeria monocytogenes*, *Staphylococcus aureus*, *Enterococcus faecalis*, and *Pseudomonas aeruginosa*	[35]
	Inhibits *Phanerochaete chrysosporium*, *Gloeophyllum trabeum*, *Penicillium purpurogenum*, *Trichoderma harzianum*, and *Aspergillus fumigatus*	[36]
	Inhibits *Colletotrichum gloeosporioides*, *Botrytis cinerea*, and *Fusarium graminearum*	[37]
Anti-inflammatory	Blocks production of interleukin (IL)-1β, IL-6, and tumor necrosis factor-alpha (TNF-α)	[14]
	Inhibits heat-induced erythrocyte hemolysis and hypotonic solution-induced erythrocyte hemolysis	[38]
	Treats allergic dermatitis, such as atopic dermatitis	[39]
Insecticidal	Insecticidal against mosquito and midge (*Chaoborus plumicornis*) larvae, cabbage white butterfly (*Pieris rapae*) larvae, termite (*Reticulitermes virginicus*), fruit fly (*Drosophila melanogaster*), and fire ant (*Solenopsis invicta × richteri*)	[10]
	Larvicide for mosquito (*Culex pipiens*) control	[24]
	Acaricidal capacity against *Tetranychus cinnabarinus*	[40]
	Strong contact toxicity against cotton aphid	[41]
Antioxidative	Free radical scavenging activity	[14,42,43]
Algicidal	Inhibits cell growth of *Microcystis aeruginosa* and *Chlamydomonas reinhardtii* Induces chlorophyll degradation and decreases algae photosynthesis	[24,44]
Allelopathic	Inhibits seed germination and seedling growth of lettuce (*Lactuca sativa*) and perennial ryegrass (*Lolium perenne*)	[10]

Chen et al. [27] demonstrated that the essential oil isolated from the leaves of *C. camphora* by hydrodistillation had effective activity against *Staphylococcus aureus* (MICs = 8.0 μg/mL), *Enterococcus faecalis* (MICs = 3.2 μg/mL), *Bacillus subtilis* (MICs = 0.8 μg/mL), *Salmonella* *enterica gallinarum* (MICs = 1.6 μg/mL), and *Escherichia coli* (MICs = 0.8 μg/mL). Similarly, Bottoni et al. [35] described the essential oil isolated from the aerial parts of the plant as showing discrete inhibitory activity toward the tested bacterial strains *S. aureus*, *E. faecalis*, *Listeria monocytogenes*, and *Pseudomonas aeruginosa* (all MIC equal to 25 mg/mL, except for the MIC against *L. monocytogenes*, which was 12.5 mg/mL). These results are supported in other work in which the leaf EOC demonstrated stronger inhibitory capacity against Gram-negative (*E. coli* and *P. aeruginosa*) than Gram-positive bacteria (*S. aureus* and *B. subtilis*) [6].

Water extracts from *C. camphora* leaves were found to be inhibited in their activity by treatment of 5% for wood fungi *Phanerochaete chryosporium*, *Gloeophyllum traveum*, *Penicillium purprogenum*, *Trichoderma harzianum* and *A. fumigatus* [36]. One wood stain fungus (*Botryodiplodia theobromae*) could also be suppressed when the concentration was increased to 10% [36]. The authors reasoned that the antimicrobial effect of the water extract could be due to its composition, which included 76.2%, D-camphor and minor constituents, such as 3-methyl-2-butenoic acid (8.6%), 1,8-cineole (4.7%) and 1,6-octadien-3-ol (4.5%).

Wang et al. [37] extracted the EOC from leaves to test its activity against plant pathogenic fungi, such as *Colletotrichum gloeosporioides*, *Botrytis cinerea*, and *Fusarium graminearum*. Notable inhibitory activity was found after 48 h of EOC treatment, with half-maximal inhibitory concentrations (IC_50_) of 31.74, 35.79, and 38.02 mg/L, for the three separate strains, highlighting the potential use of EOC as a natural preservative for fruits and vegetables. It has been suggested that EOC could be used in the preparation of a strong fungistatic agent against *C. cucurbitarum* infection. Pragadheesh et al. [11] emphasized the inhibitory action of (1*R*)-(+)-camphor against the growth of *Choanephora cucurbitarum*, a wet rot pathogen of *Withania somnifera*. Furthermore, in comparison to (1*R*)-(+)-camphor, *C. camphora* oil revealed superior activity. Fungal growth inhibition by (1*R*)-(+)-camphor and plant essential oil was due to cytoplasm coagulation and hyphal lysis of *C. cucurbitarum.*

Some researchers observed a synergistic antimicrobial effect of constituents of EOC [23,45]. Poudel et al. [23] suggested that the antimicrobial effect of the wood EOC against *S. marcescens* could be due to synergism among the major constituents of the EOC (camphor, 1,8-cineol, α-terpinol, and safrole) and other components. Viljoen et al. showed that a combination of 1,8-cineole and camphor produced a synergistic interaction and improved the antimicrobial effect on *Candida albicans* [45].

### 4.2. Anti-Inflammatory Activities

EOCs have long been prescribed in traditional medicine for the treatment of inflammation-related diseases, such as rheumatism, bronchitis and muscle pains. Lee et al. [13] reported that the ethanol extract of *C. camphora* blocked the production of interleukin (IL)-1β, IL-6, and tumor necrosis factor-α (TNF-α) from RAW264.7 cells stimulated by lipopolysaccharide. Another study suggested EOC as a natural treatment for skin inflammation and the possibility of applying medicinal plants to treat various other inflammation-related diseases. Xiao et al. [38] investigated the anti-inflammatory activity of borneol-type EOC in vitro (human erythrocyte membrane stability assay) and in vivo (acute inflame murine model). The essential oil nanoemulsion inhibited heat-induced erythrocyte hemolysis (IC_50_ = 5.29 mg/mL) and hypotonic solution-induced erythrocyte hemolysis (IC_50_ = 0.26 mg/mL). Moreover, both single and repeated topical administration of the EOC on mice auricles reduced xylene-induced auricle swelling. This anti-inflammatory action of *C. camphora* is known to be due to cytokine secretion and control of macrophage-mediated inflammation (IL-1β, IL-6, and TNF-α).

Fu et al. [42] described the amelioration of oxidative stress and inflammation in diet-induced rats treated with *C. camphora* seed kernel oil. This treatment reduced the level of inflammatory markers by increasing the activity of serum glutamate oxaloacetate transaminase and glutamate-pyruvate transaminase and peroxisome proliferator-activated receptor gamma (PPAR-α). Kang et al. [39] described the efficacy of EOC from leaves in treating allergic inflammation, such as atopic dermatitis. The extract demonstrated a notable anti-inflammatory effect in human adult low-calcium high-temperature keratinocytes, and ameliorative effects on 2,4-dinitrochlorobenzene-induced atopic dermatitis in mice. These findings will facilitate the development of EOC as a new and natural therapeutic agent for inflammatory skin conditions.

### 4.3. Insecticidal and Acaricidal Activities

Essential oil-based pesticides have a wide range of pest management applications with several advantages, such as being readily available, renewable, and readily degraded to minimize environmental side effects [10]. Currently, there is increasing use of plant-derived essential oils as insecticides and repellents in agriculture and the health sector.

The EOC has been found to have a certain degree of mosquito repellence. Xu et al. [24] confirmed the potent larvicidal efficacy of EOC against the mosquito *Anopheles stephensi*. Satyal et al. [10] examined the insecticidal activity of leaf EOC against mosquito (*Culex pipiens*) and midge (*Chaoborus plumicornis*) larvae, cabbage white butterfly (*Pieris rapae*) larvae, termites (*Reticulitermes virginicus*), fruit flies (*Drosophila melanogaster*), and red imported fire ants (*Solenopsis invicta × richteri*). The oil showed mosquito and midge larvicidal activities but was most effective against cabbage butterfly larvae, fruit flies, and fire ants (median lethal concentration (LC_50_) = 186, 153, and 176 μg/mL, respectively).

Chen and Dai [40] reported that the ethanol extract of *C. camphora* displayed remarkable acaricidal activity against the mite *Tetranychus cinnabarinus*. The most active constituents of the extract were 2,4-di-*tert*-butylphenol and ethyloleate, with LC_50_ values of 1850.94 and 2481.65 mg/kg, respectively, after a seven-day treatment in a potted seedling experiment.

Jiang et al. [41] reported the insecticidal potential of seed, leaf, and twig EOC against cotton aphids. The seed EOC exhibited the highest repellent activity (89.86%, after 24 h of treatment at a concentration of 20 μL/mL). The LC_50_ values of 245.79, 274.99, and 146.78 mg/L were reported for the three essential oils after 48 h of treatment, respectively. Linalool was a major contributor to the insecticidal and repellent effects [46].

### 4.4. Antioxidative Activities

As alluded to above, *C. camphora* seed kernel oil increased the antioxidative activity and lowered the concentration of malondialdehyde (a biomarker of lipid peroxidation and oxidative stress) in diet-induced rats by increasing the concentration of superoxide dismutase and catalase [42]. Liu et al. [43] demonstrated the in vitro antioxidative property of the flavonoids extracted from *C. camphora* leaves. The flavonoids exhibited a dose-dependent increase in the antioxidant activity, as measured by the 1,1-diphenyl-2-picryl-hydrazyl (DPPH) free radical scavenging assay and the ferric reducing antioxidant power assay, with remarkable results compared to commercial antioxidants. Similar findings were reported by Lee et al. [13]. Most of the antioxidative/free radical scavenging effect was observed in the butanol extract (in the DPPH assay) and the ethanol extract (in the xanthine oxidase activity assay) of *C. camphora*, with IC_50_ values of 14 and 15 μg/mL, respectively.

### 4.5. Allelopathic and Algicidal Activities

Allelochemicals from plants are considered effective, economic, and environmentally friendly herbicides and algaecides due to their effective inhibitory action against herbs and algae, convenient preparation, and easy degradation in nature. Satyal et al. [9] evaluated the allelopathic activities of Nepalese *Cinnamomum* essential oils in terms of inhibition of seed germination, as well as inhibition of seedling growth against a representative dicot (lettuce, *Lactuca sativa*) and a representative monocot (perennial ryegrass, *Lolium perenne*). *Lactuca sativa* seed germination was notably inhibited by EOC (IC_50_ = 149 μg/mL), as well as its major component, camphor (IC_50_ = 239 μg/mL). Furthermore, the EOC inhibited the seedling growth of *L. perenne* and *L. sativa*, with relatively greater efficacy against *L. sativa.*

A number of studies have shown the potential value of *C. camphora* as an algaecide. Yakefu et al. [44] observed the inhibitory effects of the water and methanol extracts, respectively, of fresh *C. camphora* leaves on *Microcystis aeruginosa* and *Chlamydomonas reinhardtii* algae cell growth by inducing chlorophyll degradation and reducing photosynthesis. Methanol extracts showed superior inhibitory effects to water extracts at the same concentration because of a greater number and higher concentration of compounds in the methanol extracts. It was thought that linalool and camphor were the major compounds responsible for the observed anti-algal activity. However, other terpenoids in the *C. camphora* extracts might also contribute to the inhibitory effects. In other studies, cell growth of the algae *Chlorella vulgaris* and *C. reinhardtii* was reduced when exposed to 1,8-cineole and limonene due to the degradation of photosynthetic pigments and decreased photosystem II efficiency [44].

## 5. Discussion

So far, we have briefly looked at the characteristics of EOC obtained from *C. camphora* and its various uses. For a long time, *C. camphora* has been used to heal human diseases and has also been used in cultural and religious fields. In recent years, the scope of use is expanding. The camphor tree has a range of applications in various fields, such as industry, cosmetics, pesticides, and pharmaceuticals.

With the development of analysis and separation/purification technology, there have been many advances in the study of the composition of EOC. However, the specific composition is not yet fully understood. In various areas, most attempts are made to utilize the EOC rather than using specific components because of the advantage of eliminating processes such as separation and purification; however, it may have disadvantages in harnessing its full potential. To realize the pharmacological activity of EOC, systematic and high-level research is needed, aimed at characterization and standardization. It is also necessary to understand the medicinal mechanism of EOC. Moreover, quality control is poorly researched, and no direct clinical evidence has been reported. Therefore, research on the in vitro and in vivo biological activity of various monomer compounds should be strengthened in the future so that plants in this genus can better contribute to human health. In addition, well-developed methods should be established to ensure the consistency, safety, and efficacy of *C. camphora*. Taken together, because of the high economic and practical value of *C. camphora*, research is needed to modernize the traditional use of the plant and increase its utilization value.

For proper use of essential oil, it is essential to consider its possible effects on humans and the environment. Natural products, such as essential oils, are a group of substances with various characteristics that can cause mutations, genotoxicity, and carcinogenicity in mammals, so the effect of essential oils on non-target creatures should also be identified. Furthermore, some constituents of EOC still need a safety risk assessment. The daily maximum human therapeutic dose of D-camphor is about 1.43 mg/kg. This dose is relatively safe, but long-term data are still lacking. Although safrole showed notable insecticidal activity, it is carcinogenic. Linalool harmed the aquatic environment. In addition, 1,8-cineol can be used as a flavoring and pharmaceutical ingredient at an appropriate level, but at high doses, it can be toxic to the respiratory and nervous system.

*C. camphora* is grown as an ornamental plant and used as a raw material for furniture and a source of camphor. Furthermore, its essential oil can be used as an alternative medicine and an important source for perfume. This study was conducted to collect information related to the phytochemistry of the genus *C. camphora* and ultimately lay the foundation for future research and development focusing on the antimicrobial and antioxidant actions and application examples, and the potential role of recent advancements in science and technology with improved efficacy and worldwide applicability. For broader utilization of *C. camphora* extracts and essential oils, it is necessary to increase efficacy and stability and reduce costs through in-depth exploration of formulation development according to the purpose of use.

## Figures and Tables

**Table 1:** Examples of volatile oil constituents from leaves of *Cinnamomum camphora*.

Constituent	Camphor-Type * [11]	Borneol-Type * [13]	Cineol-Type * [24]	Linalool-Type * [27]
α-Pinene	3.7	8.3	4.7	2.2
Camphene	2.2	4.3	0.2	0.5
α-Thujene	0.2	0.5	-	
Sabenene	0.5	1.1	17.4	
β-Pinene	1.4	2.2	3.4	0.7
α-Phellandrene	0.7	0.7	-	5.1
*p*-Mentha-2,4(8)-diene	1.9	-	-	
*o*-Cymene	0.2	0.1	-	-
*m*-Cymene	-	-	-	
1,8-Cineole	1.1	4.6	53.5	16.8
α-*trans*-Ocimene	0.1	-	-	0.5
γ-Terpinolene	0.1	-	-	0.5
2,2,5-Trimethylhexane-3,4-dione	-	-	-	
4,7-Dimethyl-4,4a,5,6-tetrahydrocyclopenta[c]pyran-1,3-dione	-	-	-	
2,5,9-Trimethyldecane	-	-	-	
Isoterpinolene	0.5	-	-	-
Linalool	-	-	-	26.6
1,3,8-*p*-Menthatriene	-	-	-	
D-Camphor	73.8	0.8	-	5.0
Terpinen-4-ol	0.8	-	-	1.1
*endo*-Borneol	0.4	66.8	0.1	8.1
α-Terpineol	-	0.4	9.5	8.7
*p*-Menth-1-en-4-ol	-	-	-	
*p*-Menth-1-en-8-ol	-	-	-	
Elixene	-	-	-	
Dihydro-*cis*-α-copaene-8-ol	-	-	-	
α-Bourbonene	-	-	-	
1,5-Dimethyl-8-isopropenyl-1,5-cyclodeca-diene	-	-	-	
Caryophyllene	-	-	1.2	3.3
Aromadendrene	-	-	-	
γ-Elemene	-	0.2	0.1	1.3
α-Caryophyllene	-	1.8	0.5	1.9
β-Selinene	-	-	1.2	
Others				17.7
Total	99.3	98.0	99.2	100

* Relative percentage (%) of compounds in volatile oils.

## References

[1] Robi A., Sujanapal P., Udayan P. (2014). *Cinnamomum agasthyamalayanum* sp. nov. (Lauraceae) from Kerala, India. Int. J. Adv. Res..

[2] Alam K., Nawab M., Kazmi M. (2019). Pharmacological and therapeutic profile of Käfür (*Cinnamomum camphora* (L.) J. Presl)—A Review. Hippocrat. J. Unani Med..

[3] Garg N., Jain A. (2017). Therapeutic and medicinal uses of Karpura-A review. Int. J. Sci. Res. (IJSR).

[4] CABI Invasive Species Compendium.

[5] Malabadi R.B., Kolkar K.P., Meti N.T., Chalannavar R.K. (2021). Camphor tree, *Cinnamomum camphora* (L.); Ethnobotany and pharmacological updates. Biomedicine.

[6] Zhou Y., Yan W. (2016). Conservation and applications of camphor tree (*Cinnamomum camphora*) in China: Ethnobotany and genetic resources. Genet. Resour. Crop Evol..

[7] Korea Ministry of Cultural Sports and Tourism. https://www.nhc.go.kr:1500/news/general.do?idx=52.

[8] Kumar S., Kumari R., Mishra S. (2019). Pharmacological properties and their medicinal uses of *Cinnamomum*: A review. J. Pharm. Pharmacol..

[9] Guo S., Geng Z., Zhang W., Liang J., Wang C., Deng Z., Du S. (2016). The chemical composition of essential oils from *Cinnamomum camphora* and their insecticidal activity against the stored product pests. Int. J. Mol. Sci..

[10] Satyal P., Paudel P., Poudel A., Dosoky N.S., Pokharel K.K., Setzer W.N. (2013). Bioactivities and compositional analyses of Cinnamomum essential oils from Nepal: *C. camphora*, *C. tamala*, and *C. glaucescens*. Nat. Prod. Commun..

[11] Pragadheesh V., Saroj A., Yadav A., Chanotiya C., Alam M., Samad A. (2013). Chemical characterization and antifungal activity of *Cinnamomum camphora* essential oil. Ind. Crops Prod..

[12] Hattori A. (2001). Camphor in the Edo era fireworks. Yakushigaku Zasshi.

[13] Shi S., Wu Q., Su J., Li C., Zhao X., Xie J., Gui S., Su Z., Zeng H. (2013). Composition analysis of volatile oils from flowers, leaves and branches of *Cinnamomum camphora* chvar. Borneol in china. J. Essent. Oil Res..

[14] Lee H.J., Hyun E.-A., Yoon W.J., Kim B.H., Rhee M.H., Kang H.K., Cho J.Y., Yoo E.S. (2006). In vitro anti-inflammatory and anti-oxidative effects of *Cinnamomum camphora* extracts. J. Ethnopharmacol..

[15] Singh R., Jawaid T. (2012). *Cinnamomum camphora* (Kapur). Pharmacogn. J..

[16] Li Q., Wang X.-X., Lin J.-G., Liu J., Jiang M.-S., Chu L.-X. (2014). Chemical composition and antifungal activity of extracts from the xylem of *Cinnamomum camphora*. BioResources.

[17] Babu K.N., Sajina A., Minoo D., John C., Mini P., Tushar K., Rema J., Ravindran P. (2003). Micropropagation of camphor tree (*Cinnamomum camphora*). Plant Cell Tissue Organ Cult..

[18] Bhavaniramya S., Vishnupriya S., Al-Aboody M.S., Vijayakumar R., Baskaran D. (2019). Role of essential oils in food safety: Antimicrobial and antioxidant applications. Grain Oil Sci. Technol..

[19] Benali T., Habbadi K., Khabbach A., Marmouzi I., Zengin G., Bouyahya A., Chamkhi I., Chtibi H., Aanniz T., Achbani E.H. (2020). GC–MS Analysis, Antioxidant and Antimicrobial Activities of *Achillea Odorata* Subsp. *Pectinata* and *Ruta Montana* Essential Oils and Their Potential Use as Food Preservatives. Foods.

[20] Burt S. (2004). Essential oils: Their antibacterial properties and potential applications in foods—A review. Int. J. Food Microbiol..

[21] Dhifi W., Bellili S., Jazi S., Bahloul N., Mnif W. (2016). Essential oils’ chemical characterization and investigation of some biological activities: A critical review. Medicines.

[22] Hyldgaard M., Mygind T., Meyer R.L. (2012). Essential oils in food preservation: Mode of action, synergies, and interactions with food matrix components. Front. Microbiol..

[23] Poudel D.K., Rokaya A., Ojha P.K., Timsina S., Satyal R., Dosoky N.S., Satyal P., Setzer W.N. (2021). The Chemical Profiling of Essential Oils from Different Tissues of *Cinnamomum camphora* L. and Their Antimicrobial Activities. Molecules.

[24] Xu Y., Qin J., Wang P., Li Q., Yu S., Zhang Y., Wang Y. (2020). Chemical composition and larvicidal activities of essential oil of *Cinnamomum camphora* (L.) leaf against Anopheles stephensi. Rev. Soc. Bras. Med. Trop..

[25] Chalchat J.-C., Valade I. (2000). Chemical composition of leaf oils of *Cinnamomum* from Madagascar: *C. zeylanicum Blume*, *C. camphora* L. *C. fragrans Baillon* and *C. angustifolium*. J. Essent. Oil Res..

[26] Stubbs B.J., Specht A., Brushett D. (2004). The essential oil of *Cinnamomum camphora* (L.) Nees and Eberm.—Variation in oil composition throughout the tree in two chemotypes from eastern Australia. J. Essent. Oil Res..

[27] Chen J., Tang C., Zhang R., Ye S., Zhao Z., Huang Y., Xu X., Lan W., Yang D. (2020). Metabolomics analysis to evaluate the antibacterial activity of the essential oil from the leaves of *Cinnamomum camphora* (Linn.) Presl. J. Ethnopharmacol..

[28] Carrubba A., Catalano C. (2009). Essential oil crops for sustainable agriculture—A review. Climate Change, Intercropping, Pest Control and Beneficial Microorganisms.

[29] Choi J.K. (1997). Longevity Way with Traditional Ethnophamacology in Korea (Tojong Yakcho Jangsubeob).

[30] Pharmacopoeia Commission of the Ministry of Health of the People’s Republic of China (2010). Pharmacopia of the People’s Republic of China.

[31] Kumar M., Ando Y. (2003). Single-wall and multi-wall carbon nanotubes from camphor—A botanical hydrocarbon. Diam. Relat. Mater..

[32] Gomes-Carneiro M.R., Felzenszwalb I., Paumgartten F.J. (1998). Mutagenicity testing of (±)-camphor, 1, 8-cineole, citral, citronellal, (-)-menthol and terpineol with the Salmonella/microsome assay. Mutat. Res. /Genet. Toxicol. Environ. Mutagenes..

[33] Donkin R.A. (1999). Dragon’s Brain Perfume: An Historical Geography of Camphor.

[34] Wang L., Zhang K., Zhang K., Zhang J., Fu J., Li J., Wang G., Qiu Z., Wang X., Li J. (2020). Antibacterial activity of *Cinnamomum camphora* essential oil on *Escherichia coli* during planktonic growth and biofilm formation. Front. Microbiol..

[35] Bottoni M., Milani F., Mozzo M., Radice Kolloffel D.A., Papini A., Fratini F., Maggi F., Santagostini L. (2021). Sub-Tissue Localization of Phytochemicals in *Cinnamomum camphora* (L.) J. Presl. Growing in Northern Italy. Plants.

[36] Wang J., Su B., Jiang H., Cui N., Yu Z., Yang Y., Sun Y. (2020). Traditional uses, phytochemistry and pharmacological activities of the genus Cinnamomum (Lauraceae): A review. Fitoterapia..

[37] Wang J., Cao X., Song L., Ding Z., Tang F., Yue Y. (2017). Comparative chemical composition and antifungal activity of the essential oils of *Cinnamomum camphora* L. Presl Leaves from three geographic origins. Food Sci..

[38] Xiao S., Yu H., Xie Y., Guo Y., Fan J., Yao W. (2021). The anti-inflammatory potential of *Cinnamomum camphora* (L.) J. Presl essential oil in vitro and in vivo. J. Ethnopharmacol..

[39] Kang N.-J., Han S.-C., Yoon S.-H., Sim J.-Y., Maeng Y.H., Kang H.-K., Yoo E.-S. (2019). *Cinnamomum camphora* leaves alleviate allergic skin inflammatory responses in vitro and in vivo. Toxicol. Res..

[40] Chen Y., Dai G. (2015). Acaricidal activity of compounds from *Cinnamomum camphora* (L.) Presl against the carmine spider mite, Tetranychus cinnabarinus. Pest Manag. Sci..

[41] Jiang H., Wang J., Song L., Cao X., Yao X., Tang F., Yue Y. (2016). GC× GC-TOFMS analysis of essential oils composition from leaves, twigs and seeds of *Cinnamomum camphora* L. Presl and their insecticidal and repellent activities. Molecules.

[42] Fu J., Zeng C., Zeng Z., Wang B., Gong D. (2016). *Cinnamomum camphora* seed kernel oil ameliorates oxidative stress and inflammation in diet-induced obese rats. J. Food Sci..

[43] Liu Z., Kong L., Lu S., Zou Z. (2019). Application of a combined homogenate and ultrasonic cavitation system for the efficient extraction of flavonoids from *cinnamomum camphora* leaves and evaluation of their antioxidant activity in vitro. J. Anal. Methods Chem..

[44] Yakefu Z., Huannixi W., Ye C., Zheng T., Chen S., Peng X., Tian Z., Wang J., Yang Y., Ma Z. (2018). Inhibitory effects of extracts from *Cinnamomum camphora* fallen leaves on algae. Water Sci. Technol..

[45] Viljoen A., Van Vuuren S., Ernst E., Klepser M., Demirci B., Başer H., Van Wyk B.-E. (2003). *Osmitopsis asteriscoides* (Asteraceae)-the antimicrobial activity and essential oil composition of a Cape-Dutch remedy. J. Ethnopharmacol..

[46] Gonzalo O.E., Raúl S., María I.B., David H.-P., Omar S.-M. (2017). Antifeedant effects of common terpenes from Mediterranean aromatic plants on *Leptinotarsa decemlineata*. J. Soil Sci. Plant Nutr..

